# Demographic and socioeconomic factors associated with cervical cancer screening among women in Serbia

**DOI:** 10.3389/fpubh.2023.1275354

**Published:** 2024-01-05

**Authors:** Slavica Djordjevic, Katarina Boricic, Snezana Radovanovic, Ivana Simic Vukomanovic, Olgica Mihaljevic, Verica Jovanovic

**Affiliations:** ^1^Department of the High School of Health, Academy of Applied Studies Belgrade, Belgrade, Serbia; ^2^Faculty of Medical Sciences, University of Kragujevac, Kragujevac, Serbia; ^3^Institute of Public Health of Serbia “Dr. Milan Jovanović Batut”, Belgrade, Serbia; ^4^Faculty of Medical Sciences, Department of Social Medicine, University of Kragujevac, Kragujevac, Serbia; ^5^Institute for Public Health, Kragujevac, Serbia; ^6^Faculty of Medical Sciences, Department of Pathophysiology, University of Kragujevac, Kragujevac, Serbia

**Keywords:** socioeconomic inequalities, Pap test, National Health Survey, Serbia, cancer screening

## Abstract

**Objectives:**

Effective reduction of cervical cancer incidence and mortality requires strategic measures encompassing the implementation of a cost-effective screening technology. Serbia has made significant strides, introducing organized cervical cancer screening in 2012. However, various impediments to screening implementation persist. The aim of the study was to estimate the socioeconomic factors associated with cervical cancer screening among women in Serbia.

**Methods:**

Data from 2019 National Health Survey of the population of Serbia were used in this study. The study is cross sectional survey on a representative sample of the population of Serbia. Present total number of participants analyzed in survey 6,747.

**Results:**

In Serbia, 67.2% of women have done a Pap test at any time during their lives, of which 46.1% of women have undergone cervical cancer screening in the past 3 years. About a quarter of women have never undergone a Pap test in their life (24.3%). The probability of never having a Pap test have: the youngest age group (15–24 years) is 1.3 times more likely than the oldest age group (OR = 1.31), unmarried women 0.3 times more often than married women (OR = 0.37), respondents with basic education 0.9 times more often than married women (OR = 0.98), the women of lower socioeconomic status 0.5 times more often than respondents of high socioeconomic status (OR = 0.56).

**Conclusion:**

Enhancement of the existing CCS would be the appropriate public health approach to decrease the incidence and mortality of cervical cancer in the Republic of Serbia.

## Introduction

Cervical cancer ranks as the fourth most prevalent malignancy among females globally, exhibiting the highest prevalence in underdeveloped and developing nations. Incidence rates, standardized by age, demonstrate considerable variation, ranging from 75 per 100,000 women in high-risk countries to fewer than 10 per 100,000 women in low-risk countries (1). Approximately 90% of cervical carcinoma-related mortalities arise in low- and middle-income countries, where the fatality rate is twice as high as in underdeveloped and developing countries (2). The global burden of cervical carcinoma is projected to escalate, reaching 700,000 cases and 400,000 deaths by 2030. These escalating figures predominantly pertain to low- and middle-income countries, posing a significant global health challenge. Addressing this disparity in cervical carcinoma incidence and mortality is of utmost importance for the global health community (3).

Data from the Cancer Registry of the Republic of Serbia in 2020 reveal a standardized incidence rate of 29.2 per 100,000 women and a standardized mortality rate of 10.7 per 100,000 women, indicating a substantial burden of cervical cancer in Serbia (4). Effective reduction of cervical cancer incidence and mortality requires strategic measures encompassing the implementation of a cost-effective screening technology (5).

Most countries’ governments have already incorporated cervical carcinoma screening into their healthcare systems (6). In this context, the Republic of Serbia has made significant strides, introducing organized cervical cancer screening in 2012, thereby expanding preventive healthcare services for women in the realm of reproductive health. Thus far a total of four 3-year-long screening cycles has been carried out in the population of women aged between 25 and 64 years. The current screening coverage for cervical cancer on the territory of Serbia varies between 35 and 68%, and it is regionally dependent. However, various impediments to screening implementation persist, primarily related to demographic, socioeconomic, and cultural factors.

Some studies explaining the socio-economic and demographic factors associated with cervical cancer screening have been recently published. Factors including lack of awareness and knowledge about cervical cancer, lack of access to information, excessive cost of cervical cancer screening, low risk perceptions, and poor health seeking behaviors were major barriers for women seeking cervical cancer screening. Social networks, socio-cultural norms, and perceptions of the role of men and stigma also engender negative attitudes and behaviors. Barriers to cervical cancer screening included poorly equipped health facilities and a lack of national cancer prevention policies and programs (7, 8).

There are also ongoing challenges related to the availability and accessibility of screening services. Promoting awareness among women is imperative to empower them to take control of their health. Access to primary healthcare is pivotal in expanding cervical carcinoma screening coverage. Collaborations with professional associations and academic institutions, civil society, non-governmental organizations, women’s groups, media, and public opinion leaders play a vital role in promoting preventive initiatives, enhancing health literacy, and removing socioeconomic barriers to screening service utilization.

Identifying socioeconomic factors influencing women’s participation in screening will enable the development of interventions to surmount these barriers, improve screening service accessibility and availability, and intensify primary and secondary prevention efforts among the target population.

The aim of the study was to determine the socioeconomic factors associated with cervical cancer screening among women in Serbia. The results of this study are expected to help decision makers, health care providers and community to design strategies, in order to effectively reduce inequalities in cervical cancer screening.

## Methods

### Study type

The study is a part of the population health research of Serbia, conducted in the period from October to December 2019 by the Institute of Statistics of the Republic of Serbia incooperation with the Institute of Public Health of Serbia “Dr. Milan Jovanović Batut” and the Ministry of Health of the Republic of Serbia. The study is cross sectional survey on a representative sample of the population of Serbia.

### Population to be included

Present total number of participants analyzed in survey 6,747.

### Collection

A two-stage stratified sample was used for the study. Data were stratified by type of settlement (urban and other settlements) and by geographical areas (Belgrade region, Vojvodina region, Šumadija region, and Western Serbia, Southern and Eastern Serbia region). The 2011 census conducted in the Republic of Serbia was used as a framework for sample selection. The sample size was calculated based on the precision requirements for the evaluation of the standard error of the indicator; “proportion of people prevented from performing daily activities”; according to the recommendations of EUROSTAT for conducting population health surveys. This research is planned to be used for obtaining statistically reliable ratings at the level of Serbia as a whole, and then at the level of four regions: Belgrade Region, Vojvodina Region, Šumadija Region, and Western Serbia, Southern, and Eastern Serbia Region, and the population of cities and other settlements. As a compromise between the required assessment accuracy and the cost of conducting the survey, a sample size of 6,000 households was established, which were expected to cover about 15,000 members aged 15 and over and about 1,500 children aged 5–14. In calculating the sample size, children ages 5–14 were not included. It was decided to select 10 households in each survey county. Reserve households were provided for each survey county in case a large number of households in the survey county refused to cooperate. By dividing the total number of households by the number of households in the sample per census district, it was calculated that 600 census districts should be selected. A sample of 5,114 households was selected, registering a total of 15,621 people, of whom 13,589 were aged 15 and older and 1,493 were children aged 5–14.

The survey was conducted over 3 months (October–December) 2019, in accordance with the recommendations of the European Health Survey—third wave, according to which the period of data collection in the field must last at least 3 months, of which atleast 1 month must be in the period September–December, i.e., in autumn.

Ethical standards in the Health Research of the Serbian population are in line with the international Declaration of Helsinki, adopted at the General Assembly of the World Medical Association in 1964, and amended in 2013, as well as with the legislation of the Republic of Serbia. In order to maintain the privacy of research participants and the confidentiality of information collected about them, all necessary steps were taken in accordance with the General Data Protection Regulation (GDPR), a new European legal framework that prescribes the handling of citizens’ personal data, as well as the National Personal Data Protection Act, the Personal Data Protection Strategy, and the Official Statistics Act, with the application of the principle of statistical confidentiality.

The existing database was transferred to the University of Kragujevac with an official letter from the Serbian Institute of Public Health of Serbia. This study was approved by the competent territorial Ethics Committees of the four main regions of Serbia with headquarters in the National Institute of Public Health in Belgrade.

### Research instrument

Standardized questionnaires constructed according to the European Health Interview Survey (EHIS, wave 3), (9) and adapted to the specific regional characteristics served as the research instrument. Three types of questionnaires and one measurement form were used:

- “Face-to-face” interviewing—recording responses to questions during oral communication between the interviewer and the respondent.
- Self-completion of the questionnaire by the respondents without the involvement of the interviewer.
- Measurement of basic anthropometric characteristics (height and body mass), and blood pressure.

In a “face-to-face” interview with an individual, the interviewer completed a structured and semi-structured research instrument (questionnaire) in the presence of the interviewee. The use of a self-completion questionnaire meant that the interviewee was given a structured questionnaire and instructions and completed it themselves, without the assistance of an interviewer. The questionnaire completed by the respondent was then passed on to the interviewer according to a predefined procedure. Computer-assisted personal interview (CAPI) and paper-and-pencil (PAPI) methods were used to complete the questionnaire.

### Variables measured in the study

The independent variables encompassed demographic: age (15–24, 25–34, 35–44, 45–54, 55–64, and 65 and more years), marital status [never (un)married community, divorce, separation, death of a partner, and marriage/non-marital union], education (primary and lower, secondary, and high), working status (unemployed, inactive, and employed), region (Belgrade, Vojvodina, Shumadia and Western Serbia, and Southern and Eastern Serbia), and self-assessment of health (bad and very bad, medium, and good and very good). The Demographic and Health Survey Wealth Index (DHS) was used as an indicator of material condition, according to which households or respondents were classified into five socio-economic categories or quintiles of the welfare index: first quintile (poorest), second quintile (poor), third quintile (middle class), fourth quintile (rich), and fifth quintile (richest). To create this index, variables related to living conditions and the possession of various durable goods were used: the number of bedrooms per household member, the material from which the floor, roof, and walls of the living space are made, the type of water supply and sanitation, the type of fuel used for heating, owning a color television, a mobile phone, a refrigerator, a washing machine, a dishwasher, a computer, air conditioning, central heating, and a car. For the purposes of this research, according to the well-being index, the respondents were divided into three categories of material status: rich (fourth and fifth quintile), middle class (third quintile), and poor (first and second quintile).

On the other hand, the dependent variable of interest was the utilization of cervical cancer screening by Papanicolaou (Pap) smear test: the frequency of the cervical cancer screening (during last 12 months, 1–2 years ago, 2–3 years ago, 3 or more years ago, and never) and upon whose initiative it was done (by own initiative, by the doctor’s advice, and by the doctor’s call within the scrining).

### Statistical methods

All data of interest were presented and analyzed by adequate mathematical-statistical methods appropriate for the data type. *χ*^2^ test was applied to test the difference in the frequency of categorical variables. Prevalence of cervical cancer screening (CCS), crude, and adjusted odds ratios (ORs) with 95% Confidence intervals (CIs) were calculated to examine demographic and socio-economic factors associated with inequalities in utilization of cervical cancer screening. All results with a probability equal to, or less than 5% (*p* ≤ 0.05) were considered statistically significant. Statistical analysis was performed using a commercial, standard software package SPSS, version 19.0 [The Statistical Package for Social Sciences software (SPSS Inc., version 19.0, Chicago, IL, United States)].

## Results

A total of 6,747 women aged 15 years and older were surveyed, average age of 52.02 ± 19.12 (min 15, max 99). The majority of the respondents were either married or living in cohabitation (58.1%), hailing from Vojvodina (31.4%), and having completed secondary education (51.3%). The highest percentage of respondents belonged to the low-income category (41.3%) (Table 1).

**Table 1:** Socio-demographic characteristics of the respondents and the frequency of the cervical cancer screening (Pap test).

Variables	*N* (%)	During last 12 months	1–2 years ago	2–3 years ago	3 or more years ago	Never	*p* ^*^
Age							
15–24	715 (10.6)	7.4	4.9	2.4	0.4	28.5	<0.001
25–34	764 (11.3)	20.1	16.1	9.4	2.5	9.6	
35–44	954 (14.1)	25.3	20.7	14.1	8.0	7.4	
45–54	992 (14.7)	20.7	21.6	17.4	13.2	7.6	
55–64	1,261 (18.7)	16.4	22.3	26.1	24.0	12.6	
≥65	2,061 (30.5)	10.2	14.4	30.6	52.0	34.3	
Marital status							
Never (un)married community	1,161 (17.2)	15.5	12.3	7.4	3.2	35.9	<0.001
Divorce, separation, and death of a partner	1,659 (24.6)	12.6	16.7	25.2	36.9	25.4	
Marriage/non-marital union	3,927 (58.1)	71.9	71.0	67.4	59.9	38.7	
Education							
Primary and lower	2,113 (31.3)	13.8	14.8	22.7	40.0	47.8	<0.001
Secondary	3,390 (50.3)	53.9	60.2	61.3	47.3	42.7	
High	1,244 (18.4)	32.3	24.9	16.0	12.7	9.5	
Working status							
Unemployed	1,140 (17.0)	19.7	22.5	18.8	11.7	17.0	<0.001
Inactive	3,482 (52.0)	27.5	32.2	47.4	68.3	67.8	
Employed	2,125 (31.0)	52.8	45.3	33.8	20.0	15.2	
Wellbeing index							
Poor	2,787 (41.3)	29.9	32.8	41.0	44.4	53.4	<0.001
Middle class	1,381 (20.5)	19.6	22.8	20.0	22.1	18.6	
Rich	2,579 (38.2)	50.5	44.4	39.0	33.5	18.0	
Region							
Belgrade	1,527 (22.6)	23.4	20.3	20.3	23.5	21.4	<0.001
Vojvodina	2,117 (31.4)	21.9	37.9	34.9	31.9	35.6	
Shumadia and Western Serbia	1,478 (21.9)	18.7	18.2	18.7	22.5	28.1	
Southern and Eastern Serbia	1,625 (24.1)	36.0	24.6	26.1	21.1	14.9	
Self-assessment of health							
Bad and very bad	1,176 (8.3)	8.4	7.6	11.6	22.6	32.1	<0.001
Medium	1,741 (27.1)	22.9	23.4	28.2	35.0	23.5	
Good and very good	3,830 (56.7)	68.7	69.0	60.2	42.4	44.4	

^*^χ^2^ test.

Approximately 67.2% of the female participants had undergone the Pap test at least once in their lifetimes. Among these, a considerable subset, accounting for 46.1%, had undergone cervical cancer screening within the preceding 3 years (21.3% within the last 12 months, and 15.4% less than 2 years ago). A total of 24.3% of women had never undergone a Pap test. The largest percentage of respondents underwent the Pap test based on the advice of their doctors (22.5%), followed by 21.3% who sought the test on their own, and only 2.2% who did so in response to a doctor’s recommendation within an organized screening program.

Over the previous 12 months, the Pap test was most commonly conducted among women aged 35–44 years (25.3%), those who were married (71.9%), residents of Southern and Eastern Serbia (36%), who had completed secondary education (53.9%), who were employed (55.8%), who belonged to the richest part of the population (50.5%), and those who perceived their health as good and very good (68.7%) (Table 1).

When analyzed by demographic and socio-economic characteristics, a statistically significant correlation was observed between all the features and the frequency of screening. Females aged 35–44 most frequently chose to undergo screening voluntarily (25.3%), while women in the age group 55–64, predominantly participated in organized cervical cancer screening (30.4%). Women falling within the secondary education category were 1.6 times more likely to undergo a screening examination voluntarily (56.1%) compared to women with the highest level of education (33.9%), while women with the lowest education levels most often participated in organized screening (21.6%). Married individuals most commonly sought advice from doctors for screening (72.5%), and 21.6% of divorced women predominantly participated within the framework of organized screening. When considering financial circumstances, in women from the most affluent category, there is a notably higher frequency of seeking out a Pap test (52.8%) on their own, in comparison to those in the least affluent category (27.6%). Conversely, an inverse correlation becomes apparent when opting for screening based on medical advice or within the context of organized screening, with the highest percentage (42.6%) being observed among women characterized by the least favorable financial status. In terms of employment status, employed women display a higher propensity for seeking a Pap test of their own initiative (52.5%), in contrast to their unemployed (19.8%) and inactive counterparts (27.7%). Women residing in Southern and Eastern Serbia predominantly undergo a Pap test through their own initiative (34.4%), whereas women from Vojvodina commonly heed the counsel of a doctor (32.4%), and those from Šumadija and Western Serbia primarily participate as part of an organized screening effort (39.2%). A substantial proportion of women, particularly those who rate their health as good or very good, opt for self-initiated Pap testing (73.7%), whereas those who perceive their health as poor or very poor more frequently participate within the context of organized screening (14.2%) (Table 2).

**Table 2:** The frequency of subjects according the cervical cancer screening (Pap test) and upon whose initiative it was done.

Variables	By own initiative	By the doctor’s advice	By the doctor’s call within the scrining	*p* ^*^
Age				
15–24	6.2	4.9	5.4	< 0.001
25–34	16.9	17.2	7.4	
35–44	25.3	18.2	18.9	
45–54	20.9	20.1	17.6	
55–64	18.6	20.9	30.4	
≥65	12.1	18.6	20.3	
Marital status				
Never (un)married community	16.7	9.8	7.4	< 0.001
Divorce, separation, and death of a partner	14.6	17.7	21.6	
Marriage/non-marital union	68.7	72.5	71.0	
Education				
Primary and lower	10.0	20.7	21.6	< 0.001
Secondary	56.1	59.3	55.4	
High	33.9	20.0	23.0	
Working status				
Unemployed	19.8	21.5	17.0	< 0.001
Inactive	27.7	37.3	40.8	
Employed	52.5	41.2	42.2	
Wellbeing index				
Poor	27.6	37.4	42.6	< 0.001
Middle class	19,9	21.3	23.6	
Rich	52.5	41.3	33.8	
Region				
Belgrade	21.4	22.1	20.9	< 0.001
Vojvodina	26.6	32.4	29.1	
Shumadia and Western Serbia	17.6	17.3	39.2	
Southern and Eastern Serbia	34.4	28.2	10.8	
Self-assessment of health				
Bad and very bad	5.2	11.5	14.2	< 0.001
Medium	21.1	26.1	34.5	
Good and very good	73.7	62.4	51.3	

^*^χ^2^ test.

In univariate linear regression model, the probability of never having a Pap test have: the youngest age group (15–24 years) is 1.3 times more likely than the oldest age group (OR = 1.31), unmarried women 0.3 times more often than married women (OR = 0.37), respondents with basic education 0.9 times more often than married women (OR = 0.98), the women of lower socioeconomic status 0.5 times more often than respondents of high socioeconomic status (OR = 0.56), women from the region of Vojvodina, 0.6 times more often than respondents from Southern and Eastern Serbia (OR = 0.60), as well as 0.9 times more often those who assess their health as very good (OR = 0.93). Regarding self-initiated pap testing, the probability of not having it: middle-aged women (45–54 years) have 1.7 times more often compared to the oldest (OR = 1.75), unmarried 0.7 times more often than married (OR = 0.79), those with low education attainment low education 1.3 times more often than high education attainment (OR = 1.33), the women of lower socioeconomic status 0.5 times more often from the respondents of high socioeconomic status (OR = 0.56), from Sumadia and Western Serbia 0.5 times more often than those from the regions of southern and eastern Serbia (OR = 0.55), and women who asses their health as bad 1.1 times more often than respondents who asses their health as good (OR = 1.16). Within the context of multivariate regression analysis, paramount predictors surface concerning women who have abstained from undergoing a Pap test, as well as those who voluntarily undertake such testing encompass age, marital status, educational level, well-being index, regional demographics, and self-appraisal of health status (Tables 3, 4).

**Table 3:** Linear regression analysis for women who never used the cervical cancer screening (Pap test) according to the demographic and socio-economic characteristics.

Variables	Univariate model		Multivariate model	
	OR (95%CI)	*p*	OR (95%CI)	*p*
Age				
15–24	1.31 (0.94–1.83)	<0.001	1.60 (0.96–2.66)	0.069
25–34	0.61 (0.41–0.90)	0.014	0.78 (0.48–1.26)	0.319
35–44	0.55 (0.36–0.84)	0.006	0.60 (0.38–0.96)	0.034
45–54	0.66 (0.43–1.02)	0.067	0.69 (0.43–1.09)	0.119
55–64	0.99 (0.66–1.48)	0.265	0.98 (0.64–1.49)	0.430
≥65	1		1	
Marital status				
Never (un)married community	0.37 (0.25–0.53)	< 0.001	0.269 (0.010–0.556)	0.042
Divorce, separation, and death of a partner	0.35 (0.25–0.49)	< 0.001	0.313 (0.058–0.601)	0.047
Marriage/non-marital union	1		1	
Education				
Primary and lower	0.98 (0.887–1.178)	< 0.001	0.41 (0.073–0.798)	0.019
Secondary	0.50 (0.388–0.658)	< 0.001	0.44 (0.301–0.643)	< 0.001
High	1		1	
Working status				
Unemployed	1.28 (0.620–2.073)	0.339	2.41 (1.067–4.011)	0.363
Inactive	0.89 (0.691–1.176)	0.164	3.35 (1.358–5.690)	0.194
Employed	1		1	
Wellbeing index				
Poor	0.56 (0.482–0.706)	< 0.001	0.45 (0.216–0.729)	< 0.001
Middle class	0.27 (0.151–0.424)	< 0.001	0.25 (0.053–0.476)	0.017
Rich	1		1	
Region				
Belgrade	0.24 (0.110–0.402)	0.001	0.23 (0.034–0.451)	0.091
Vojvodina	0.61 (0.506–0.776)	< 0.001	0.76 (0.502–1.091)	< 0.001
Shumadia and Western Serbia	0.58 (0.469–0.763)	< 0.001	0.62 (0.320–0.991)	< 0.001
Southern and Eastern Serbia	1		1	
Self-assessment of health				
Bad and very bad	0.93 (0.873–1.093)	< 0.001	1.12 (0.972–1.389)	< 0.001
Medium	0.23 (0.151–0.343)	< 0.001	0.23 (0.084–0.401)	0.003
Good and very good	1		1	

^*^Reference category: never had a Pap test.

**Table 4:** Linear regression analysis for used the cervical cancer screening (Pap test) own initiative according to the demographic and socioeconomic characteristics.

Variables	Univariate model		Multivariate model	
	OR (95%CI)	*p*	OR (95%CI)	*p*
Age				
15–24	1.48 (1.13–1.95)	0.519	1.381 (1.09–2.16)	0.956
25–34	0.83 (0.67–1.18)	<0.001	0.919 (0.54–1.54)	0.028
35–44	1.15 (0.75–2.43)	<0.001	0.972 (0.34–2.71)	0.065
45–54	1.75 (0.68–2.94)	<0.001	1.667 (0.62–3.00)	0.089
55–64	1.85 (0.93–2.77)	<0.001	1.740 (0.96–3.13)	0.151
≥65	1		1	
Marital status				
Never (un)married community	0.80 (0.72–0.96)	< 0.001	0.45 (0.25–0.71)	< 0.001
Divorce, separation, death of a partner	0.85 (0.79–0.99)	< 0.001	0.50 (0.251–0.80)	< 0.001
Marriage/non-marital union	1		1	
Education				
Primary and lower	1.34 (1.29–1.53)	< 0.001	1.11 (0.95–1.37)	0.005
Secondary	0.37 (0.28–0.50)	< 0.001	0.55 (0.14–1.03)	0.015
High	1		1	
Working status				
Unemployed	1.78 (1.39–2.37)	0.160	3.17 (1.27–5.40)	0.224
Inactive	3.21 (2.30–4.45)	0.248	3.16 (1.78–4.87)	0.363
Employed	1		1	
Wellbeing index				
Poor	0.56 (0.49–0.69)	< 0.001	0.48(0.28–0.73)	< 0.001
Middle class	0.25 (0.15–0.38)	< 0.001	0.22 (0.04–0.42)	0.048
Rich	1		1	
Region				
Belgrade	0.40(0.30–0.55)	< 0.001	0.29 (0.13–0.50)	0.005
Vojvodina	0.42 (0.32–0.56)	< 0.001	0.31(0.16–0.50)	< 0.001
Shumadia and Western Serbia	0.56 (0.46–0.71)	< 0.001	0.36 (0.15–0.62)	< 0.001
Southern and Eastern Serbia	1		1	
Self-assessment of health				
Bad and very bad	1.16 (1.10–1.34)	< 0.001	1.16 (0.99–1.44)	< 0.001
Medium	0.49 (0.42–0.62)	< 0.001	0.45 (0.30–0.64)	< 0.001
Good and very good	1		1	

^*^Reference category: self-initiated Pap test.

## Discussion

While the occurrence of cervical cancer is diminishing in developed nations due to robust screening and vaccination initiatives, the ailment persists as a significant contributor to cancer-related morbidity and mortality in regions constrained by limited resources. The accessibility of technologies for averting cervical cancer remains uneven, laboratories frequently lack adequate infrastructure to employ them, and HPV awareness is not universally disseminated. The consensus within the scientific community is that this disease is eminently preventable, offering a potent opportunity for successful intervention when identified during precancerous and early malignant stages. Swift and effective treatment delivery is crucial for ensuring a favorable prognosis (10).

Countries adeptly executing organized screening initiatives exhibit a pronounced reduction in both the incidence and mortality rates of cervical cancer. Their experiences can serve as instructive paradigms for nations yet to institute organized programs or those grappling with suboptimal levels of execution and quality. Primarily encompassing underdeveloped and developing countries, where exposure to risk factors remains conspicuously elevated and preventive endeavors are limited, the burden of cervical cancer continues to linger at a significant threshold (11).

Low-to-middle income countries, burdened with a heightened prevalence of cervical cancer, persistently confront financial and logistical constraints in ensuring the availability of both cervix cancer screening and human papillomavirus vaccines to their populace. The pivotal challenge lies in orchestrating screening program strategies attuned to the unique circumstances of these nations, thus attaining widespread coverage within the target demographic through assessments of appropriate efficacy. Such endeavors are paramount for wresting control over the escalating trajectory and for achieving the stipulated decline in both incidence and mortality rates over the forthcoming decades (12).

In August 2020, the 73rd World Health Assembly supported the WHO Global Strategy to Accelerate the Elimination of Cervical Cancer as a Public Health Problem 2020–2030 (WHO, 2018, 2020). Achieving the strategy goals by 2030 could reduce incidence rates from the current average of 13.3 to less than 4 new cases per 100,000 women to ensure that cervical cancer is no longer a worldwide public health issue. Interventions need to be strategically designed on three main pillars with a comprehensive approach: immunization, screening, and disease treatment. The recommended strategy (90–70-90) is to vaccinate 90% of girls under the age of 15 with the HPV vaccine, screen 70% of women at least twice, once before the age of 35 and again before the age of 45 with an appropriate test, and treat 90% of premalignant and invasive malignant lesions, which will lead to decrease incidence rates of cervical cancer about 42% by 2,045 and 97% by 2,120, saving about 62 million human lives cumulatively (13).

In spite of the endeavors pursued by the international community, a multitude of investigations have substantiated the persistence of several impediments to cervical cancer screening. These barriers encompass a spectrum of sociodemographic determinants, encompassing elements such as awareness, attitudes, beliefs, perceived risk, psychological considerations, self-efficacy, prior experiences, temporal constraints, household dynamics, cultural influences, fatalistic outlooks, social support networks, access discrepancies, cost considerations, safety concerns, insurance parameters, and the overall healthcare framework (14, 15).

Consequently, a study conducted among a cohort of Czech women illuminated that predominant factors underpinning the non-attendance of screening initiatives included the absence of discernible symptoms, apprehension toward the procedural aspect, and anxiety regarding a prospective cancer diagnosis (16).

An additional study revealed that, despite possessing a commendable level of cervical cancer knowledge and maintaining a favorable stance regarding screening, the actual implementation of screening remained limited due to the imposition of social stigma. Findings stemming from an inquiry conducted among a cohort of Indian women demonstrated that 43.64% exhibited a constructive disposition toward screening, 20.31% were familiar with screening via the PAP test, and a mere 13.22% engaged in the practice of screening (17). The results of our research showed that approximately 67.2% of the participants had undergone a Pap test at least once in their life. Among them, 21.3% in the last 12 months, and 15.4% less than 2 years ago. A total of 24.3% of women had never undergone a Pap test. The largest percentage of respondents did the Pap test based on the doctor’s advice (22.5%), followed by 21.3% who requested the test themselves and only 2.2% who did it in response to a doctor’s recommendation within an organized screening program.

A survey conducted on a sample of Hungarian women aged between 25 and 65 years revealed that 74% had undergone screening examinations within the preceding 3 years, either as part of or outside an organized screening program. More than half of the target population had not sought information about cervical cancer screening at all. Among those who sought screening information, the majority obtained it from gynecologists, while one-third obtained it from media sources and health service brochures, and 21% from general practitioners. Furthermore, the results indicated that general practitioners had been successful in motivating women who initially declined to participate in the screening program. A significant portion of Hungarian women had not been informed about cervical cancer screening beyond the invitation letter, with only 35.3% of women aged 25–65 being invited to organized cervical cancer screening (18).

The challenges in achieving comprehensive screening coverage, even within nations with established national cervical cancer screening programs, are highlighted by the findings of the National Health Survey conducted in Japan. The survey revealed that screening rates for cervical cancer are notably low, ranging from 15.1% for women aged 20–24 years to 49.4% for women aged 30–34 years, with an overall screening rate across all age groups of 2.1% (19).

In spite of the establishment of Estonia’s national cervical cancer (CC) screening program in 2006, the CC incidence within the country remained among the highest in Europe by 2020. The lifetime uptake prevalence of Pap smears witnessed a notable escalation from 50.6% in 2004 to 86.7% in 2020 (20).

A vast majority (93%) of American women report undergoing at least one Pap smear within their lifetime. Among women without a history of abnormal smears, 55% engage in annual Pap smear screenings, 17% opt for a biennial screening interval, and16% adhere to triennial screenings, while 11% do not partake in regular screenings. Remarkably, even among the older adult population, a considerable proportion engages in frequent screening—38% of women aged 75–84, and 20% of women aged 85 and older reported undergoing annual Pap smears (21).

High levels of education, employment, and stable interpersonal relationships are positively associated with Papanicolaou (Pap) test utilization among women in Brazil. Data pertaining to Papanicolaou test performance and socio-economic variables were gathered from 559 women in Mato Grosso do Sul (MS), as well as 338 women in Paraíba (PB). Among women in PB with a low educational level and unemployment status, the chances of having undergone the Papanicolaou test ≥ three times, or once within the last 3 years, were 2.96- and 2.40-fold lower, respectively. Also, the odds of women in MS who were not in a stable relationship to have undergone the test ≥ three times were 1.79-fold lower compared to women in stable relationships (22).

In Denmark, 74% of women participated in organized cervical cancer screening (23). Similar results have been observed in our study, indicating that 67.2% of women have undergone the Pap test at some point in their lives, of whom 46.1% have undergone cervical cancer screening in the past 3 years. Moreover, a slight increase in the percentage of women undergoing the Pap test has been noted compared to the results of the National Health Survey conducted in 2013 (24), suggesting intensified efforts to enhance screening coverage in Serbia. Nonetheless, demographic and socioeconomic barriers identified in 2013 (24) still persist, underscoring the necessity of studies of this nature to identify these factors and formulate strategies for mitigating the identified obstacles.

Systemic, personal, and cultural barriers, as well as the lack of decision-making guidelines, are also contributors to disparities in cervical cancer screening in numerous countries such as Latin America (25), sub-Saharan Africa (26), and Thailand, where reasons for non-screening include shyness and time constraints (27). Literature reviews reveal diverse barriers to accessing healthcare services at large, along with specific hindrances encompassing sociodemographic factors such as age, education, employment, and marital status, cultural distinctions, past traumatic personal experiences, and healthcare worker competencies. These complexities contribute to varying effects on women’s participation in cervical cancer screening (28).

Thus, considerable efforts are warranted to augment women’s participation in cervical cancer screening. Healthcare systems must reinforce resources to meet the evolving needs. Achieving screening program efficiency necessitates the enhancement of educational interventions, professional and interprofessional collaboration, and the formulation of health and social policies targeted at barrier elimination. Women’s education about the importance of cancer screening is imperative. General practitioners could play a pivotal role in mobilizing women to utilize preventive services. Involving general practitioners in organizing cervical cancer screening programs could increase participation rates among women who typically decline services.

Strategies and requisite interventions should be devised to support vulnerable groups, explore barriers among women in screening utilization, and mitigate disparities in preventive examination usage. The significance of this study lies in its endeavor to enlighten decision-makers in the Republic of Serbia’s public health domain that, despite efforts to enhance screening coverage, it remains suboptimal, necessitating ongoing education and awareness campaigns regarding the importance of preventive examinations.

Our study has several limitations: cross-sectional design, which does not permit inferences about potential causal relations between the explanatory variables and disorders of interest, and self-reporting, which is always prone to recall biases in describing. Further research in the field is also needed in order to explore longitudinal trends and identify other potential factors of inequalities in cervical cancer screening.

## Conclusion

Implementation of population-wide health education programs and enhancement of the existing CCS would be the appropriate public health approach to decrease the incidence and mortality of cervical cancer in the Republic of Serbia. The ongoing CCS program, established in accordance with EU regulations and in line with WHO recommendations, is a good starting point for developing the necessary strategy to meet the aforementioned long-term goals. Facilitating active engagement of the vulnerable female demographic in screening and ensuring the protection of their reproductive health requires collaborative efforts across various sectors, encompassing both healthcare and non-healthcare domains, as well as active involvement from civil society.

## Data availability statement

The original contributions presented in the study are included in the article/supplementary material, further inquiries can be directed to the corresponding author.

## Ethics statement

Ethical standards in Health Research of the Serbian population are in accordance with the international Declaration of Helsinki, as adopted at the General Assembly of the World Medical Association in 1964 and amended in 2013, as well as with the legislation of the Republic of Serbia. In order to maintain the privacy of research participants and the confidentiality of information collected about them, all necessary steps were taken in accordance with the General Data Protection Regulation (GDPR), a new European legal framework that prescribes the handling of citizens’ personal data, as well as the National Personal Data Protection Act, the Personal Data Protection Strategy, and the Official Statistics Act, with the application of the principle of statistical confidentiality. Number of ethical approval is 7703/1, from the 8th December 2021, issued by the ethical committee of the Institute of Public Health of Serbia.

## Author contributions

SD: Conceptualization, Data curation, Formal Analysis, Investigation, Methodology, Project administration, Resources, Validation, Writing – original draft, Writing – review & editing, Visualization. KB: Conceptualization, Data curation, Formal Analysis, Investigation, Methodology, Project administration, Validation, Visualization, Writing – review & editing, Supervision. SR: Conceptualization, Data curation, Formal Analysis, Methodology, Supervision, Validation, Writing – review & editing. IS: Conceptualization, Data curation, Formal Analysis, Methodology, Supervision, Validation, Writing – review & editing, Visualization, Writing – original draft. OM: Conceptualization, Data curation, Supervision, Validation, Writing – review & editing. VJ: Conceptualization, Data curation, Supervision, Validation, Writing – review & editing, Formal Analysis, Funding acquisition, Investigation, Methodology, Project administration, Resources, Writing – original draft.
